# Supplementary material for: Trichoderma virens and Pseudomonas chlororaphis Differentially Regulate Maize Resistance to Anthracnose Leaf Blight and Insect Herbivores When Grown in Sterile versus Non-Sterile Soils
Source: Plants (Basel). 2024 Apr 30;13(9):1240. doi: 10.3390/plants13091240 (PMC11085588; doi:10.3390/plants13091240)
Supplement: Supplementary file 1 [file plants-13-01240-s001.zip › plants-2939259-supplementary.pdf]

Supplemental Table S1. List of metabolites of phytohormone and oxylipin profiling and the abbreviations

Compound	Chemical/Common Name
9-HOD	9(*S*)-hydroxy-10(*E*),12(*Z*)-octadecadienoic acid
9-HOT	9(*S*)-hydroxy-10(*E*),12(*Z*),15(*Z*)-octadecatrienoic acid
9,10-EpOM	cis-9,10-expoxy-12(*Z*)-octadecenoic acid **/** Coronaric acid
9,10-EpOD	cis-9,10-expoxy-12(*Z*),15(Z)-octadecadienoic acid
9,10-diHOM	threo-9,10-dihydroxy-12(*Z*)-octadecenoic acid
9-KOD	9(*S*)-oxo-10(*E*),12(*Z*)-octadecadienoic acid
9-KOT	9(*S*)-oxo-10(*E*),12(*Z*),15(*Z*)-octadecatrienoic acid
9,10-KOMA	9-hydroxy-10-oxo-12(*Z*)-octadecenoic acid
9,10-KODA	9-hydroxy-10-oxo-12(*Z*),15(*Z*)-octadecadienoic acid
13,10-KOMA	13-hydroxy-10-oxo-11(*E*)-octadecenoic acid
13,10-KODA	13-hydroxy-10-oxo-11(*E*),15(*Z*)-octadecadienoic acid
10-OPDA	10-oxo-11(*Z*),15(*Z*)-phytodienoic acid
9,12,13-THOM	9(*S*),12(*S*),13(*S*)-trihydroxy-10(*E*)-octadecenoic acid
9,12,13-THOD	9(*S*),12(*S*),13(*S*)-trihydroxy-10(*E*),15(*Z*)-octadecadienoic acid
9,10,11-THOM	9(*S*),10(*S*),11(R)-trihydroxy-12(Z)-octadecenoic acid
9,10,11-THOD	9(*S*),10(*S*),11(R)-trihydroxy-12(Z),15(*Z*)-octadecadienoic acid
AZA	nonanedicarboxylic acid **/** azelaic acid
13-HOD	13(*S*)-hydroxy-9(*Z*),11(*E*)-octadecadienoic acid
13-HOT	13(*S*)-hydroxy-9(*Z*),11(*E*),15(*Z*)-octadecatrienoic acid
12,13-EpOM	cis-12,13-expoxy-9(*Z*)-octadecanoic acid
12,13-EpOD	cis-12,13-expoxy-9(*Z*),15(Z)-octadecadienoic acid
12,13-diHOME	threo-12,13-dihydroxy-9(*Z*)-octadecenoic acid
13-KOD	13-oxo-9(*Z*),11(*E*)-octadecadienoic acid
13-KOT	13-oxo-9(*Z*),11(*E*),15(Z)-octadecatrienoic acid
OTD	13-oxo-9(*Z*),11(*E*)-tridecadienoic acid
TDD	9(*Z*),11(*E*)-tridecadiendioic acid
9,12-KOMA	9-hydroxy-12-oxo-10(*E*)-octadecenoic acid
9,12-KODA	9-hydroxy-12-oxo-10(*E*),15(*Z*)-octadecadienoic acid
13,12-KOMA	13-hydroxy-12-oxo-9(*Z*)-octadecenoic acid
13,12-KODA	13-hydroxy-12-oxo-9(*Z*),15(*Z*)-octadecadienoic acid
12-OPDA	12-oxo-10(*Z*),15(*Z*)-phytodienoic acid
dn12-OPDA	dinor-12-oxo-10(Z),15(Z)-phytodienoic acid
OPC-4:0	(Z)-4-[3-oxo-2-(pent-2-en-1-yl)cyclopentyl]butanoic acid
JA	(+)-7-iso-jasmonic acid
JA-Val	(+)-7-iso-jasmonic acid valine
JA-Leu	(+)-7-iso-jasmonic acid luecine
JA-Ile	(+)-7-iso-jasmonic acid isoluecine
12OH-JA	12-hydroxy-jasmonic acid
12OH-JA-Ile	12-hydroxy-jasmonic acid isoluecine
12COOH-JA-Ile	12-carboxy-jasmonic acid
CCA	curcurbic Acid
DH-JA	9,10-dihydro-jasmonic acid
9,10,13-THOM	9(*S*),10(*S*),13(*S*)-trihydroxy-11(*E*)-octadecenoic acid
9,10,13-THOD	9(*S*),10(*S*),13(*S*)-trihydroxy-11(*E*),15(*Z*)-octadecadienoic acid
9OH-TAN	9-hydroxy-12-oxo-10(*E*)-dodecenoic acid **/** 9-hydroxy-traumatin
9Z-TAN	12-oxo-9(*Z*)-dodecenoic acid
TA	2(*E*)-dodecenedioic acid **/** Traumatic acid
TAN	12-oxo-10(*E*)-dodecenoic acid **/** Traumatin
2OH-PA	2-hydroxy-hexadecanoic acid **/** 2-hydroxy-palmitic acid
10-HOD	10-hydroxy-8(*E*),12(*Z*)-octadecadienoic acid

2-HOT	2-hydroxy-9(*Z*),12(Z),15(*Z*)-octadecadienoic acid
CA	trans-cinnamic acid
COUMA	coumaric acid
BA	benzoic acid
SA	salicylic acid
ABA	abscisic acid
